# Choriocapillaris Alterations in Type 1 and Type 2 Torpedo Maculopathy: A Multimodal Imaging Case Series

**DOI:** 10.3390/jcm15218467

**Published:** 2026-10-30

**Authors:** Gilda Cennamo, Michele Rinaldi, Fabiana Iorio, Davide Tramontano, Erica Caporale, Ciro Costagliola

**Affiliations:** Eye Clinic, Department of Neurosciences, Reproductive Sciences and Dentistry, University of Naples Federico II, 80131 Naples, Italy; fabiana.iorio099@gmail.com (F.I.); davidetramontano40@gmail.com (D.T.); ericaporale@hotmail.it (E.C.); ciro.costagliola@unina.it (C.C.)

**Keywords:** torpedo maculopathy, optical coherence tomography angiography, choriocapillaris, retinal pigment epithelium, multimodal imaging, fundus autofluorescence, case report

## Abstract

**Background:** Torpedo maculopathy is an uncommon, usually asymptomatic chorioretinal lesion temporal to the fovea. Optical coherence tomography (OCT) distinguishes a non-cavitary type 1 from a cavitary type 2 phenotype, but choriocapillaris involvement in these phenotypes remains incompletely defined. This report describes the choriocapillaris findings in one patient with each phenotype. **Methods:** Two women with unilateral torpedo maculopathy, aged 18 (type 1) and 52 years (type 2), underwent multicolor imaging, fundus autofluorescence, spectral-domain OCT, and OCT angiography, which were assessed qualitatively. **Results:** Best-corrected visual acuity was 20/20 in both eyes of each patient. Both lesions showed outer-retinal changes and topographic correspondence between fundus autofluorescence abnormalities and choriocapillaris flow-signal attenuation. In the type 1 lesion, the inner retinal layers were preserved without cavitation, and choriocapillaris attenuation was focal and relatively homogeneous. The type 2 lesion showed subretinal cavitation (subretinal cleft), focal attenuation of the retinal pigment epithelium–Bruch membrane complex, and a choriocapillaris flow deficit that appeared more extensive within the imaged region. **Conclusions:** These observations suggest involvement of the retinal pigment epithelium–choriocapillaris unit in both phenotypes. As they derive from one patient per phenotype at a single time point, they cannot be extended to the two subtypes; longitudinal imaging is required to define the temporal relationship between outer-retinal and choriocapillaris changes.

## 1. Introduction

Torpedo maculopathy (TM) is a rare, typically unilateral and asymptomatic lesion characterized by a horizontally oval or torpedo-shaped area of hypopigmentation temporal to the fovea, with its tapered edge directed toward the foveola [1,2,3]. Although the lesion has traditionally been considered a developmental abnormality of the retinal pigment epithelium (RPE), its pathogenesis remains uncertain. Proposed mechanisms include incomplete differentiation of the fetal temporal bulge, developmental abnormalities along the horizontal raphe, and primary alteration of the RPE–choroid complex [1,3,4].

Optical coherence tomography (OCT) has expanded the phenotypic characterization of TM. Wong et al. distinguished type 1 lesions, in which outer-retinal attenuation occurs without cavitation, from type 2 lesions, characterized by cavitation or a subretinal cleft; a less common type 3 pattern with choroidal excavation has subsequently been described [3,5]. Optical coherence tomography angiography (OCTA) may demonstrate an altered flow signal at the level of the choriocapillaris, suggesting that the choroidal microvasculature may be involved together with the RPE and outer retina [6,7,8,9]. Giannakaki-Zimmermann et al. reported focal choriocapillaris attenuation in four patients with type 1 or type 2 TM, with a lesion size on OCTA comparable to that on FAF [6]. Rickmann et al. compared type 1 and type 2 TM, mainly with fundus photography, OCT and FAF; OCTA was available for two patients and showed a rarefied choriocapillaris [10].

We report two further cases, one of each phenotype, imaged with the same multimodal protocol, and describe the topographic relationship between structural OCT, fundus autofluorescence (FAF), choroidal morphology, and choriocapillaris flow signal on OCTA, with particular attention to possible OCTA artifacts.

## 2. Case Presentations

### 2.1. Imaging Protocol

All imaging was performed with the Spectralis HRA + OCT (Heidelberg Engineering, Heidelberg, Germany; Heidelberg Eye Explorer version 1.10.4.0). Multicolor imaging and blue-light FAF were acquired with the 30° objective in Case 1 and with the 55° wide-field objective in Case 2, because the lesion was located more peripherally. Structural spectral-domain OCT consisted of horizontal B-scans through the lesion (30° line scan, ART 76, quality 34 dB, in Case 1; 30° volume scan of 43 B-scans, ART 15, quality 32 dB, in Case 2). OCTA was acquired over a 10° × 10° area (approximately 3 × 3 mm) with projection-artifact removal enabled. In Case 2, the OCTA field did not include the entire lesion, and OCTA findings therefore refer to the imaged region only. All structural and OCTA scans had a quality index above 30 dB. In each patient, OCTA of the affected eye was acquired twice, and the scan with the higher quality index was analysed. The choriocapillaris was analysed on a 30-µm-thick slab located beneath Bruch membrane, with identical settings in both eyes; slab placement was verified on B-scans with flow overlay, on which the slab boundaries are displayed as red dashed lines. For reproducibility, the corresponding Heidelberg Eye Explorer settings were as follows: BM, −39 µm; distance, 70 µm; thickness, 30 µm; and contrast, 1:4. The structural en face image at the same depth was exported for each case and compared with the OCTA slab to assess shadowing. Automated segmentation was reviewed on the B-scans, and no manual correction was required.

### 2.2. Case 1: Type 1 Torpedo Maculopathy

**Figure 1:**
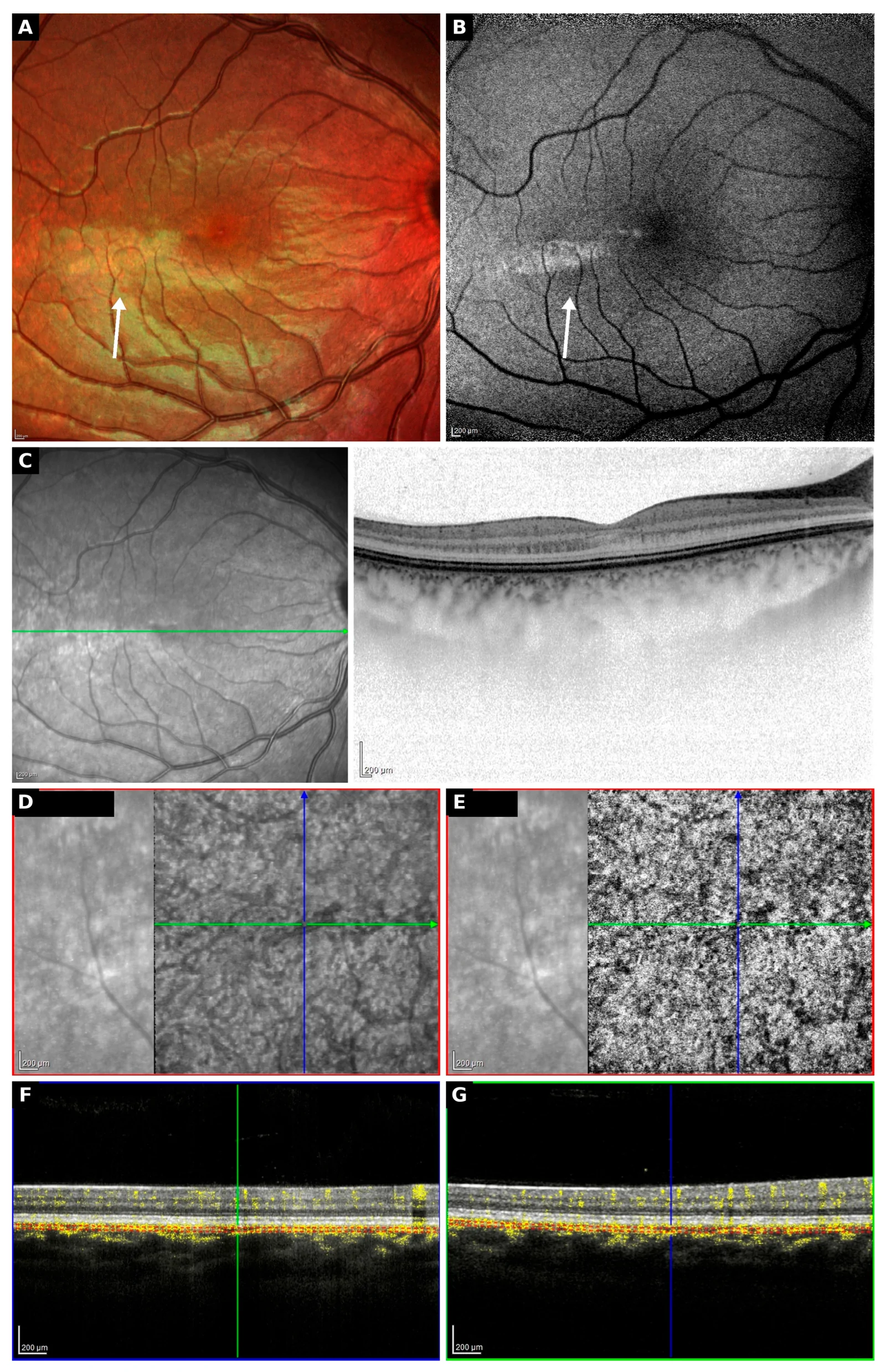
Case 1, type 1 torpedo maculopathy (right eye). (**A**) Multicolor imaging (30°) shows a well-demarcated temporal torpedo-shaped lesion (arrow). (**B**) Blue-light fundus autofluorescence (FAF) shows a mottled pattern of mildly increased autofluorescence within the lesion (arrow). (**C**) Infrared reflectance with the scan position (green line) and the corresponding spectral-domain OCT B-scan (inverted grey scale), showing subtle outer-retinal changes without cavitation, with larger choroidal vessels close to the RPE–Bruch membrane complex. (**D**–**G**) OCTA, 10° × 10° scan, 30-µm-thick choriocapillaris slab beneath Bruch membrane (Heidelberg Eye Explorer settings: BM, −39 µm; distance, 70 µm; projection-artifact removal, on): structural en face image at the same depth (**D**); en face OCTA showing focal choriocapillaris flow-signal attenuation (**E**); vertical (**F**) and horizontal (**G**) B-scans with flow overlay (yellow) and slab boundaries (red dashed lines), consistent with correct slab placement. In (**D**,**E**), the blue and green lines indicate the positions of the vertical (**F**) and horizontal (**G**) B-scans. Scale bars: 200 µm.

An asymptomatic 18-year-old woman was referred after the incidental detection of a unilateral temporal macular lesion in the right eye. Best-corrected visual acuity was 20/20 in both eyes. Anterior-segment examination was unremarkable, and no vitreous cells or other signs of intraocular inflammation were observed. She reported no relevant systemic or ocular history and no ocular trauma or surgery. The fellow eye showed no TM lesion. Refractive error and axial length were not recorded, and no follow-up imaging was available. Multicolor imaging showed a well-demarcated, torpedo-shaped lesion temporal to the fovea, with its nasal tip directed toward the foveola (Figure 1A). Blue-light FAF showed a mottled pattern of mildly increased autofluorescence within the lesion (Figure 1B).

Spectral-domain OCT showed subtle changes of the outer retinal layers within the lesion (Figure 1C). The inner retinal layers were preserved, and no cavitation or subretinal cleft was present. Beneath the lesion, the inner choroid appeared less defined, and the larger choroidal vessels appeared closer to the RPE–Bruch membrane complex than in the adjacent choroid.

OCTA showed focal, relatively homogeneous attenuation of the choriocapillaris flow signal within the lesion (Figure 1E). The structural en face image at the same depth showed no corresponding area of reduced reflectivity, and the B-scans with segmentation boundaries were consistent with correct slab placement (Figure 1D,F,G).

### 2.3. Case 2: Type 2 Torpedo Maculopathy

**Figure 2:**
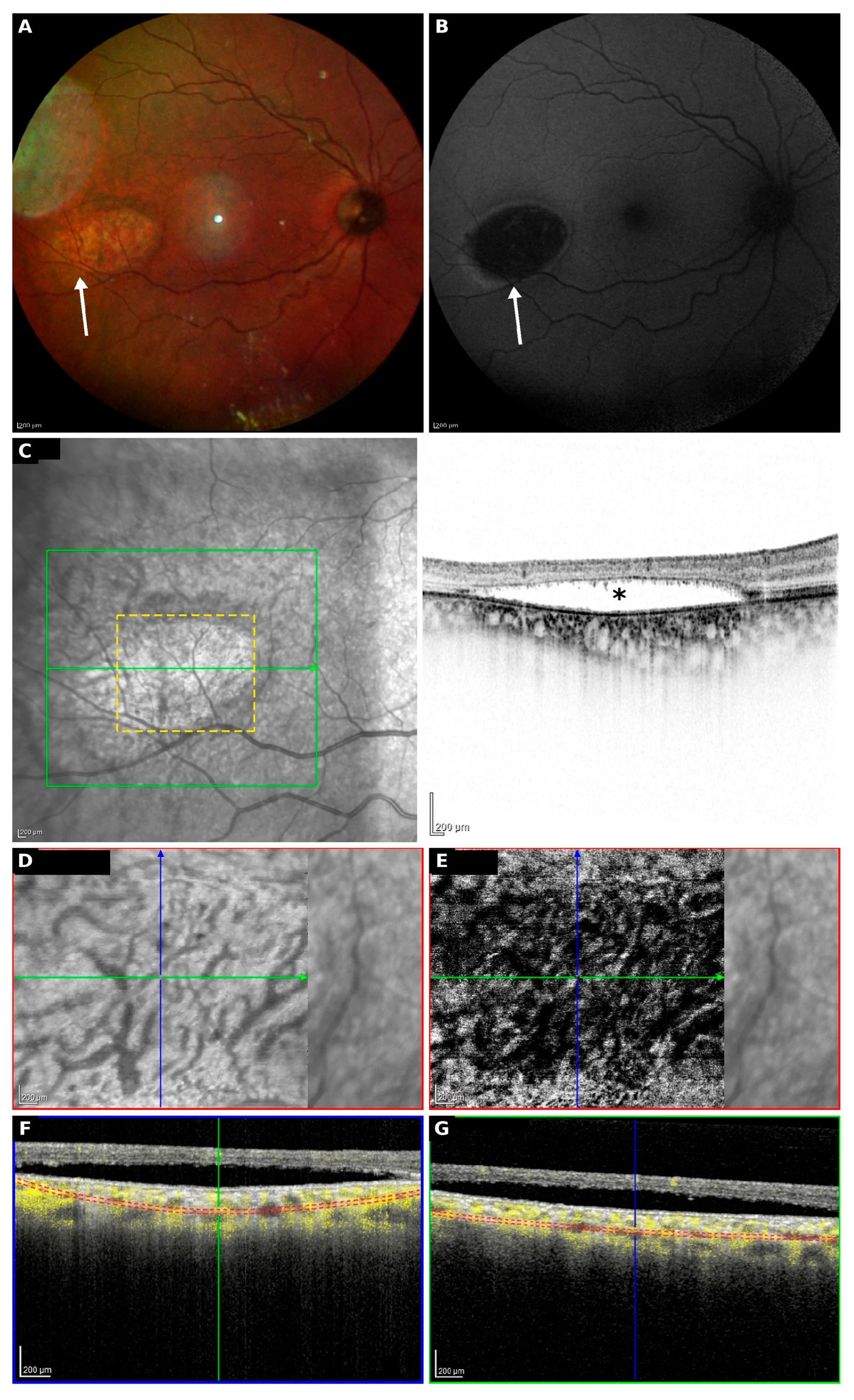
Case 2, type 2 torpedo maculopathy (right eye). (**A**) Multicolor imaging (55°) shows the temporal lesion with pigmentary remodeling (arrow). (**B**) FAF (55°) shows irregular hypoautofluorescence corresponding to the lesion (arrow). (**C**) Infrared reflectance showing the scan position (green line) and the approximate OCTA scan area of (**D**,**E**) (yellow dashed box), with the corresponding spectral-domain OCT B-scan (inverted grey scale), showing outer-retinal thinning, subretinal cavitation (subretinal cleft, asterisk) and attenuation of the RPE–Bruch membrane complex, with larger choroidal vessels beneath the lesion. (**D**–**G**) OCTA, 10° × 10° scan, 30-µm-thick choriocapillaris slab beneath Bruch membrane (Heidelberg Eye Explorer settings: BM, −39 µm; distance, 70 µm; projection-artifact removal, on): structural en face image at the same depth, showing increased reflectivity with visible large choroidal vessels (**D**); en face OCTA showing extensive choriocapillaris flow-signal attenuation within the imaged region, with sparse residual detectable flow signal toward the edges of the scan (**E**); the OCTA field did not include the entire lesion; vertical (**F**) and horizontal (**G**) B-scans with flow overlay (yellow) and slab boundaries (red dashed lines), consistent with correct slab placement. In (**D**,**E**), the blue and green lines indicate the positions of the vertical (**F**) and horizontal (**G**) B-scans. Scale bars: 200 µm.

A 52-year-old asymptomatic woman was evaluated for a unilateral torpedo-shaped lesion in the right eye. Best-corrected visual acuity was 20/20 bilaterally. The anterior segment was unremarkable, with no vitreous cells or clinical evidence of inflammation. She reported no relevant systemic or ocular history and no ocular trauma or surgery. The fellow eye showed no TM lesion. Refractive error and axial length were not recorded, and no follow-up imaging was available. Multicolor imaging demonstrated a temporal torpedo-shaped lesion with pigmentary remodeling (Figure 2A). FAF showed irregular hypoautofluorescence corresponding to the lesion (Figure 2B).

Spectral-domain OCT disclosed more pronounced outer-retinal thinning and disruption of the ellipsoid and interdigitation zones. Subretinal cavitation (subretinal cleft), corresponding to the outer-retinal cavitation of type 2 TM [3], and focal attenuation of the RPE–Bruch membrane complex were present (Figure 2C). Larger choroidal vessels were visible beneath the lesion, close to the attenuated RPE–Bruch membrane complex.

Within the imaged region, OCTA demonstrated an extensive reduction in choriocapillaris flow signal, with sparse residual detectable flow signal toward the edges of the scan (Figure 2E). The structural en face image at the same depth showed increased reflectivity with visible large choroidal vessels, and the B-scans with segmentation boundaries were consistent with correct slab placement (Figure 2D,F,G).

## 3. Discussion

In these two cases, choriocapillaris flow-signal attenuation was observed in both the non-cavitary and the cavitary lesion. The type 1 lesion showed a localized and relatively homogeneous flow-signal reduction without cavitation. The type 2 lesion combined subretinal cavitation and focal RPE–Bruch membrane attenuation with a flow-signal deficit that, within the imaged region, appeared more extensive on qualitative assessment. With one patient per phenotype, this observation cannot be extended to the two subtypes.

Previous OCTA studies have reported reduced vascular density, altered choriocapillaris texture, or focal flow deficits within TM lesions [6,7,8,9]. Rickmann et al. compared type 1 and type 2 TM in eight patients and found no age difference between the two phenotypes; OCTA, available in two patients, showed a rarefied choriocapillaris [10]. Giannakaki-Zimmermann et al. also described focal choriocapillaris attenuation in both phenotypes, with a lesion size comparable on OCTA and FAF [6]. Our observations are consistent with these reports. The additional contribution of the present cases is limited to the documentation of both phenotypes with the same protocol, including the structural en face image at the slab depth and B-scans with flow overlay to assess shadowing, and to the description of choroidal morphology beneath each lesion. Importantly, OCTA signal attenuation should not be equated automatically with anatomic vascular loss. Signals may be affected by RPE transmission changes, shadowing, projection, segmentation errors, and the detection threshold of the device. We therefore compared each en face OCTA slab with the en face structural image at the same depth and with the corresponding B-scans. No corresponding area of reduced structural reflectivity was observed within either lesion, and, in Case 2, increased reflectivity was consistent with hypertransmission through the attenuated RPE. A contribution of signal attenuation from the overlying outer retina cannot be fully excluded. Even with these precautions, histologic interpretation is not possible from OCTA alone, and the term “flow-signal attenuation” is preferable to “choriocapillaris absence.”

The qualitative topographic correspondence between the FAF abnormality and the OCTA abnormality is consistent with combined involvement of the RPE–choriocapillaris unit. FAF primarily reflects fluorophores within the RPE and is influenced by both loss of RPE cells and optical masking; consequently, it cannot be considered a surrogate measurement of choriocapillaris flow [11]. Nevertheless, concordant FAF and OCTA abnormalities may help define lesion extent and guide longitudinal image registration.

Local choroidal morphology also differed between the cases. In the type 1 lesion, the inner choroid appeared less defined, with larger choroidal vessels closer to the RPE–Bruch membrane complex. In the type 2 lesion, larger choroidal vessels were juxtaposed with an attenuated RPE–Bruch membrane complex. Similar choroidal abnormalities have been reported in multimodal studies of TM [8,12]. These descriptive findings do not imply a pachychoroid-spectrum disorder [13].

The differential diagnosis includes congenital hypertrophy of the RPE, chorioretinal scars, choroidal nevus and RPE lesions associated with familial adenomatous polyposis [10]. The characteristic shape, the temporal location with the tip directed toward the foveola, and the absence of inflammatory signs supported the diagnosis of TM in both patients.

A central limitation is that the two phenotypes were observed in different patients of different ages at a single time point. Rickmann et al. found no age difference between type 1 and type 2 TM [10]. TM is generally considered congenital and may remain stable; phenotype differences may instead reflect distinct developmental expressions. Longitudinal studies with repeatable OCTA acquisition, quantitative flow-deficit analysis, correction for signal strength and segmentation, and choroidal thickness measurements are needed to determine whether choriocapillaris changes remain stable or evolve over time. The two lesions also differed in size: on FAF and infrared images, their horizontal extent was roughly 2 mm in Case 1 and 4 mm in Case 2. These values are only approximate, as manual delineation of the lesion borders is operator-dependent, and were not used for quantitative comparison. Moreover, multicolor and FAF images were acquired with different objectives (30° and 55°); the extent of flow-signal attenuation was therefore assessed only qualitatively and refers to these two individual lesions. The small number of cases, the qualitative nature of the OCTA comparison, the incomplete OCTA coverage of the type 2 lesion, the lack of OCTA of the fellow eyes, and the absence of refractive error, axial length and follow-up data further limit generalizability.

## 4. Conclusions

In these two cases, choriocapillaris flow-signal attenuation was observed in both a type 1 and a type 2 TM lesion and, within the imaged region, appeared more extensive in the type 2 lesion. Its spatial relationship with FAF and outer-retinal abnormalities supports assessment of the RPE–choriocapillaris unit as part of multimodal characterization. These descriptive observations do not establish systematic differences between phenotypes, phenotypic progression or a primary vascular mechanism.

## Data Availability

All data relevant to the cases are included in the article. Additional clinical data are not publicly available to protect patient confidentiality.
